# Correction: Community engagement to increase vaccine uptake: Quasi-experimental evidence from Islamabad and Rawalpindi, Pakistan

**DOI:** 10.1371/journal.pone.0316862

**Published:** 2024-12-30

**Authors:** Mujahid Abdullah, Taimoor Ahmad, Ayesha Khan, Twangar Kazmi, Faisal Sultan, Sabeen Afzal, Rana Muhammad Safdar, Adnan Ahmad Khan

Ayesha Khan should be included in the author byline. Ayesha Khan should be listed as the third author, and affiliated with #1: Akhter Hameed Khan Foundation, Islamabad, Pakistan. The contributions of this author are as follows: formulated research goals, applied formal techniques to analyze and synthesize data, secured financial support, conducted research and investigation process, developed the methodology, managed and coordinated the research activity planning and execution, provided study materials, led the research activity planning and execution, verified the results or experiments, wrote the initial draft, and prepared the critical review and revision.

The correct citation is: Abdullah M, Ahmad T, Khan A, Kazmi T, Sultan F, Afzal S, et al. (2022) Community engagement to increase vaccine uptake: Quasi-experimental evidence from Islamabad and Rawalpindi, Pakistan. PLOS ONE 17(12): e0274718. https://doi.org/10.1371/journal.pone.0274718.
