# Supplementary figures and images for: Community engagement to increase vaccine uptake: Quasi-experimental evidence from Islamabad and Rawalpindi, Pakistan
Source: PLoS One. 2022 Dec 1;17(12):e0274718. doi: 10.1371/journal.pone.0274718 (PMC9714835; doi:10.1371/journal.pone.0274718)

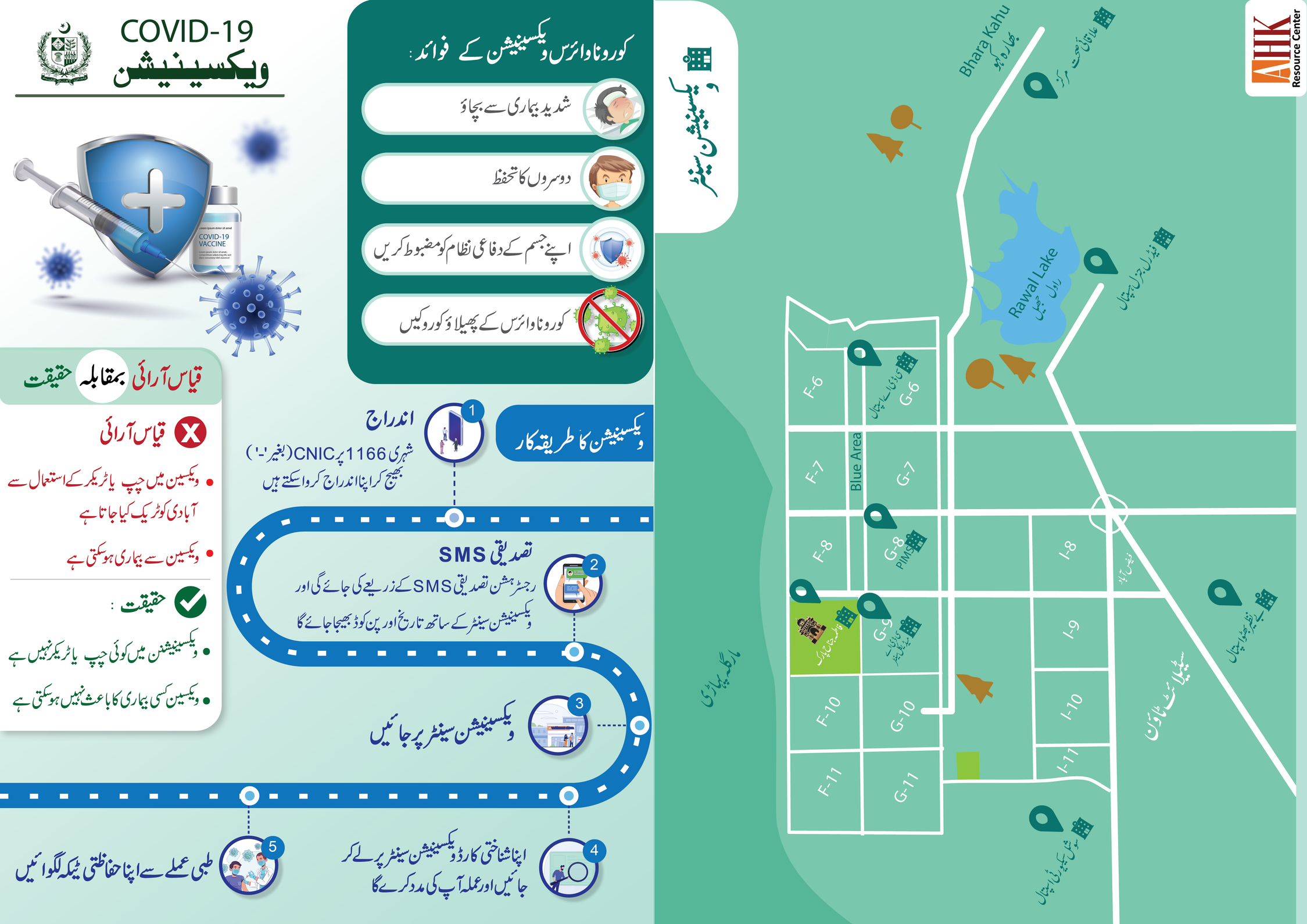

Supplement: S1 Fig — (TIF) [file pone.0274718.s001.tif]

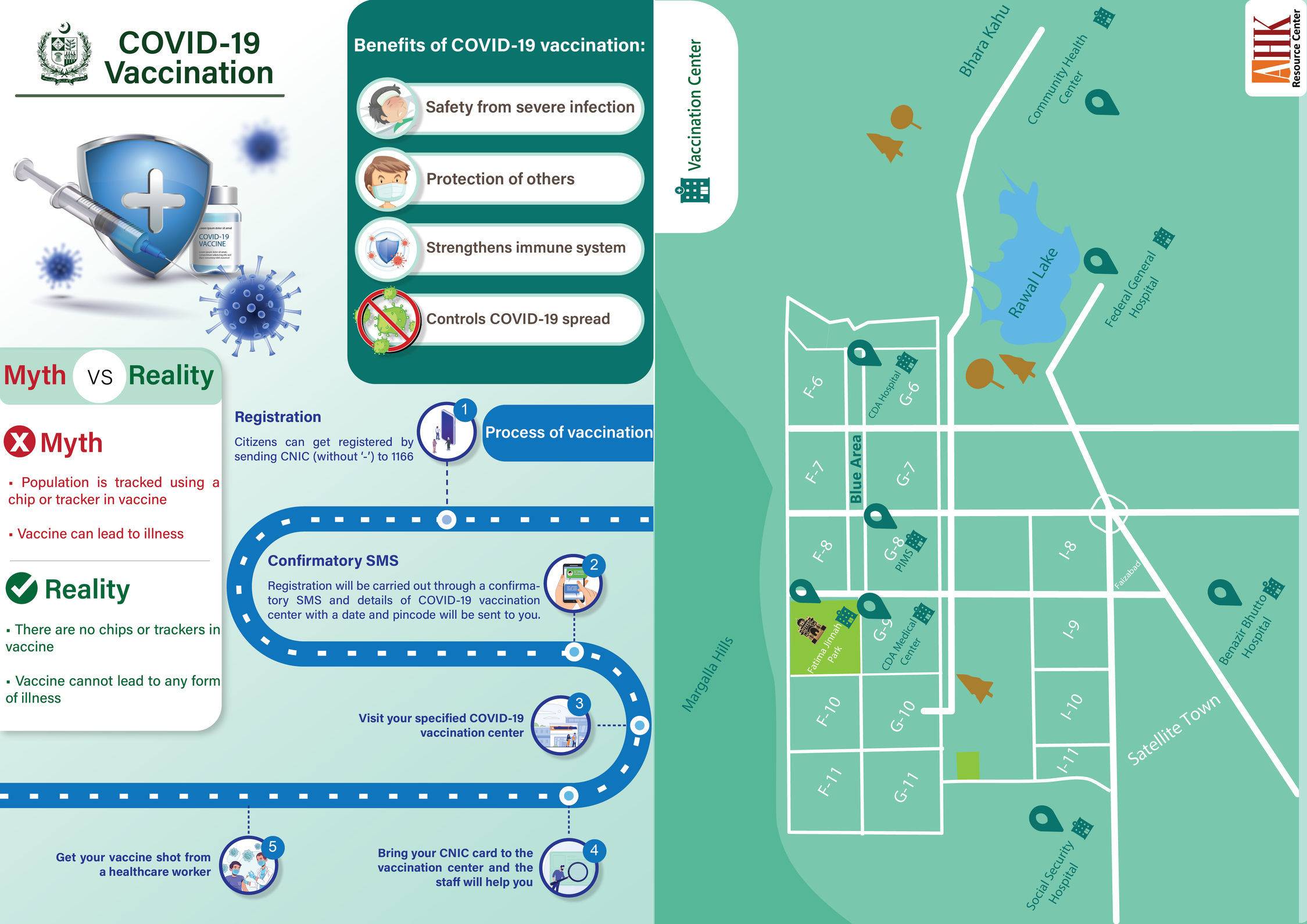

Supplement: S2 Fig — (TIF) [file pone.0274718.s002.tif]
