# Supplementary material for: Community engagement to increase vaccine uptake: Quasi-experimental evidence from Islamabad and Rawalpindi, Pakistan
Source: PLoS One. 2022 Dec 1;17(12):e0274718. doi: 10.1371/journal.pone.0274718 (PMC9714835; doi:10.1371/journal.pone.0274718)
Supplement: S1 Table — (PDF) [file pone.0274718.s003.pdf]

S1 Table. Names, descriptions and coding of variables

Variable Name	Description	Coding
**Female**	Binary variable indicating the gender of the respondent	Male = 1 (base category)<br>Female= 2
**Age Group**	Variable depicting age through five categories	18-29 (base category)<br>30-39<br>40-49<br>50-59<br>60-69
**Education Level**	Education level of respondent divided into three categories	No Education= 0 (base category)<br>Up to 12 years of education= 1<br>University degree= 2
**Ethnicity**	Ethnicity of respondent divided into five heads	Punjabi= 1<br>Pushto= 2<br>Urdu Speaking= 3<br>Hindko= 4<br>Others= 5 (base category)
**Employment Status**	Variable representing the employment status of each person in the sample	Self Employed= 1<br>Employed= 2<br>Unemployed= 3 (base category)
**Self-Infection of COVID-19**	Binary variable showing history of own infection of COVID-19	Yes = 1<br>No = 2 (base category)
**Family Infection of COVID-19**	Dummy variable showing if any member of family ever got infected with COVID-19	Yes = 1<br>No = 2 (base category)
**Family Vaccination**	Binary variable telling if any member of the household received at least one dose of COVID-19 vaccination	Yes = 1<br>No = 2 (base category)<br>Not Applicable = 3
**Risk Perception of COVID-19**	Variable indicating how worried respondents are about contracting COVID-19	Worried = 1<br>Uncertain = 2<br>Not Worried = 3 (base category)
**Television**	Binary variable if source of information regarding COVID-19 vaccination is television	No = 0 (base category)<br>Yes = 1
**Government Call/SMS**	Binary variable if source of information regarding COVID-19 vaccination is government call or sms	No = 0 (base category)<br>Yes = 1
**Family/Friends**	Binary variable if source of information regarding COVID-19 vaccination is family and friends	No = 0 (base category)<br>Yes = 1
**Medical Professional**	Binary variable if source of information regarding COVID-19 vaccination is medical professional	No = 0 (base category)<br>Yes = 1
**Religious Leader**	Binary variable if source of information regarding COVID-19 vaccination is religious leader	No = 0 (base category)<br>Yes = 1
**Distance form CVC**	Categorical variable representing the distance of respondent from a COVID-19 vaccination center	Less than 2 Km = 1<br>More than 2 Km = 2<br>Don’t Know = 3
**Any NGO/CBO working in the area**	Binary variable indicating if not even a single NGO, CBO, private charitable organization or Government welfare institutes is working in the area.	No = 0<br>Yes = 1 (base category)
**Sought treatment for last illness**	Dummy variable if respondent have received treatment for their last illness	Yes = 1<br>No = 2 (base category)
