# Supplementary material for: Community engagement to increase vaccine uptake: Quasi-experimental evidence from Islamabad and Rawalpindi, Pakistan
Source: PLoS One. 2022 Dec 1;17(12):e0274718. doi: 10.1371/journal.pone.0274718 (PMC9714835; doi:10.1371/journal.pone.0274718)
Supplement: S2 Table — (PDF) [file pone.0274718.s004.pdf]

S2 Table. Adjusted odds ratios for willingness to vaccinate and vaccine uptake

VARIABLES	(1)<br>Willingness to vaccinate	(2)<br>Vaccine uptake
**Treatment Group (C: I-10)**		
T1: G-7/F-7	0.497**	0.976
	(0.277, 0.893)	(0.644, 1.478)
T2: Bhara Kahu	0.487***	0.615**
	(0.311, 0.761)	(0.410, 0.924)
T3: Dhok Hassu	0.587**	0.445***
	(0.364, 0.945)	(0.293, 0.678)
**Period (Pre-intervention)**		
Post-intervention	1.000	1.982***
	(0.591, 1.692)	(1.231, 3.191)
**Difference-in-differences**		
T1 x Post-intervention	1.741	2.083**
	(0.791, 3.832)	(1.130, 3.839)
T2 x Post-intervention	2.061**	1.415
	(1.012, 4.195)	(0.761, 2.630)
T3 x Post-intervention	2.049*	1.620
	(0.995, 4.221)	(0.878, 2.989)
**Female**	0.514***	0.901
	(0.365, 0.722)	(0.679, 1.195)
**Age Group (18-29)**		
30-39	1.524***	1.762***
	(1.150, 2.020)	(1.339, 2.317)
40-49	2.043***	4.184***
	(1.508, 2.767)	(3.110, 5.630)
50-59	2.023***	6.474***
	(1.329, 3.081)	(4.686, 8.945)
60-69	2.525***	10.13***
	(1.507, 4.231)	(6.869, 14.94)
**Education level (None)**		
Up to 12 years	1.097	0.902
	(0.828, 1.454)	(0.700, 1.163)
University degree	1.261	1.249
	(0.840, 1.891)	(0.872, 1.788)
**Ethnicity (Others)**		
Punjabi	1.020	1.059
	(0.695, 1.498)	(0.757, 1.480)
Pushto	0.999	0.789
	(0.645, 1.549)	(0.528, 1.179)
Urdu Speaking	1.020	1.751**
	(0.507, 2.052)	(1.077, 2.847)
Hindko	1.047	1.039
	(0.509, 2.156)	(0.577, 1.871)
**Employment status (Unemployed)**		
Self-employed	1.459**	1.194
	(1.001, 2.126)	(0.865, 1.649)
Employed	2.187***	2.625***
	(1.527, 3.133)	(1.945, 3.543)
**Self-infection of COVID-19**	0.903	1.626**
	(0.470, 1.732)	(1.041, 2.541)
**Family infection of COVID-19**	3.079***	1.559**
	(1.751, 5.415)	(1.080, 2.249)

**Family vaccination (No)**		
Yes	3.254***	6.606***
	(2.436, 4.346)	(4.959, 8.799)
Not applicable	0.477***	1.232
	(0.305, 0.745)	(0.632, 2.398)
**Risk perception of COVID-19 (Unworried)**		
Worried	3.437***	1.349**
	(2.598, 4.547)	(1.042, 1.746)
Uncertain	1.443*	1.676**
	(0.946, 2.201)	(1.113, 2.523)
**Source of information on COVID-19 vaccine**		
Television	1.116	0.929
	(0.860, 1.448)	(0.739, 1.169)
Government Call/SMS	1.428**	1.154
	(1.071, 1.905)	(0.932, 1.429)
Family/friends	1.048	0.729***
	(0.813, 1.350)	(0.593, 0.896)
Medical professionals	1.455	1.292*
	(0.885, 2.390)	(0.982, 1.702)
Religious leaders	0.856	1.502
	(0.318, 2.305)	(0.860, 2.623)
**Distance from CVC (Do not know)**		
Less than 2 kms	1.483**	2.684***
	(1.091, 2.016)	(1.964, 3.667)
2+ kms	2.101***	2.368***
	(1.563, 2.825)	(1.752, 3.199)
**Any NGO/CBO working in area**	1.208	1.384***
	(0.944, 1.545)	(1.102, 1.739)
**Sought treatment for last illness (Yes)**	1.617***	1.183
	(1.217, 2.147)	(0.917, 1.526)
**Residual ICC of Clusters (Groups)**	0.0458	0.0485
	(0.0145, 0.1355)	(0.0214, 0.106)
**Constant**	0.384**	0.00882***
	(0.184, 0.802)	(0.00431, 0.0181)
**Observations**	2,904	3,216
**Number of clusters**	220	220
**Intra-class correlation**	0.046	0.048
**McKelvey & Zavoina R2 (FE and RE)**	0.395	0.533

Robust standard errors, CI eform in parentheses
*** p<0.01, ** p<0.05, * p<0.1
