# Supplementary material for: Community engagement to increase vaccine uptake: Quasi-experimental evidence from Islamabad and Rawalpindi, Pakistan
Source: PLoS One. 2022 Dec 1;17(12):e0274718. doi: 10.1371/journal.pone.0274718 (PMC9714835; doi:10.1371/journal.pone.0274718)
Supplement: S1 Questionnaire — (PDF) [file pone.0274718.s006.pdf]

Testing the Effectiveness of Community Engagement Approach on Increasing Uptake of COVID-19 Vaccination

Field	Question	Answer
consent *(required)*	AOA, my name is ______. I am from "Akhter Hameed Khan Resource Center (AHKRC)", a research organization, located at Islamabad, to assist in the implementation of socio-development programs in the country. We conduct different types of surveys and at present we are conducting a survey on "Effectiveness of Community Engagement Approach on Increasing Uptake of COVID-19 Vaccination", in your community. We would very much appreciate your participation in this survey. I would like to ask you about your knowledge of COVID-19 and its preventive vaccine.<br>اسلام علیکم، میرا نام ___________ ہے. میرا تعلق "اختر حمید خان ریسورس سینٹر" سے ہے جو کہ اسلام آباد میں واقع ایک تحقیقاتی ادارہ ہے جو ملک میں سماجی ترقی کے پروگراموں کے عمل میں مدد کرتا ہے. ہم مختلف قسم کے سروے کرتے ہیں اور اس وقت ہم "کووڈ -19 کی ویکسینیشن پر کمیونٹی کی شمولیت کی حکمت عملی کے اثرات" پر ایک سروے کر رہے ہیں. ہم اس سروے میں آپ کی شرکت کو بہت سراہتے ہیں. میں آپ سے کووڈ-19 اور اسکی حفاظتی ویکسینیشن سے متعلق آپکی معلومات کے بارے میں پوچھنا چاہتا / چاہتی ہوں.<br>The information, you will provide will be fully confidential and will be used in research purpose only. Whatever information you provide will be kept strictly confidential, will not be shown to other persons, and your name and identity will not be disclosed anywhere.<br>آپ کی فراہم کردہ معلومات کسی اور کو نہیں دکھائی جائیں گی اور آپ کے نام اور شناخت کو بھی مکمل طور پر خفیہ رکھا جائے گا. ان معلومات کو صرف تحقیق کے مقصد کیلئے استعمال کیا جائے گا.<br>Your opinion and cooperation are very important for this study. Whether you give this interview or not, totally depends on your personal will. If you are unwilling to answer any of the questions or feel embarrassed to answer, you may stop the interview any time you want or you can refrain from answering that specific question(s).<br>اس تحقیق کیلئے آپ کی رائے اور تعاون بہت اہم ہے. آپ یہ انٹرویو دیتے ہیں یا نہیں، مکمل طور پر آپ کی مرضی پر منحصرہے. اگر آپ کسی سوال کا جواب دینے کے لئے تیار نہیں ہیں یا ہچکچاہٹ محسوس کرتے ہیں تو، آپ کسی بھی وقت انٹرویو کو روک سکتے ہیں یا آپ اس مخصوص سوال کا جواب دینے سے انکار کر سکتی ہیں.<br>Your answer will be totally voluntary. The survey usually takes between 15 to 20 minutes to complete. It will be really helpful for us if you kindly spare some of your valuable time for this interview.<br>آپ کا جواب مکمل طور پر رضاکارانہ ہو گا. اس سوالنامے کو مکمل ہونے میں تقریبا 15 سے 20 منٹ لگیں گے. ہم آپ کے بہت مشکور ہونگے اگر آپ اپنے قیمتی وقت میں سے تھوڑا وقت اس انٹرویو کیلئے دیں.<br>In case of any querries or complain please contact on this number +92 51 8436877<br>کسی قسم کی معلومات یا شکایت کے لیے اس نمبر پر رابطہ کریں +92 51 8436877	1 Yes ہاں<br>2 No نہیں<br>3 Do not believe in Corona Virus نہیں میں کرونا وائرس کو نہیں مانتا
G1<br>*Group relevant when: selected(${consent} , '1')*		
G1 > Socio-demography		
Q1.1 *(required)*	Q1.1 Name of the Interviewer<br>انٹرویو لینے والے کا نام	
Q1.2 *(required)*	Q1.2 Date of the Interview<br>انٹرویو کی تاریخ	
coord *(required)*	Coordinates<br>*GPS coordinates can only be collected when outside.*	
Q1.2.1 *(required)*	Q1.2.1 Location	1 I-10<br>2 G-7 (66 Quarters)<br>3 F-7 (France Colony)<br>4 Bhara Kahu<br>5 Dhok Hassu
Q1.3 *(required)*	Q1.3 Respondent Name (Optional)<br>نام (اختیاری)	
Q1.4 *(required)*	Q1.4 Age<br>عمر	
Q1.5 *(required)*	Gender<br>جنس	1 Male مرد<br>2 Female عورت<br>96 Other: Please specify دیگر وضاحت کریں
Q1.5a *(required)*	Q1.5a Other (Please specify)<br>دیگر وضاحت کریں<br>*Question relevant when: selected(${Q1.5} , '96')*	
Q1.6 *(required)*	Q1.6 Ethnicity<br>قومیت	1 Punjabi پنجابی<br>2 Pushto پشتون<br>3 Sindhi سندھی<br>4 Afghan افغان<br>5 Balochi بلوچی<br>6 Siraiki سرائیکی<br>7 Kashmiri کشمیری<br>96 Other: Please specify دیگر وضاحت کریں<br>98 Don't know نہیں معلوم
Q1.6a *(required)*	Q1.6a Other (Please specify)<br>دیگر وضاحت کریں<br>*Question relevant when: selected(${Q1.6} , '96')*	

**Field**	**Question**	**Answer**
Q1.7 *(required)*	Q1.7 Education<br>تعلیم	1 None بالکل نہیں<br>2 Primary (6 years or less) (پرائمری (چھے سال یا اس سے کم<br>3 Secondary (7 to 10 years) (سیکنڈری (سات سے دس سال<br>4 Higher Secondary (11 to 12 years) ہائر سیکنڈری (گیارہ سے بارہ سال)<br>5 Bachelor (BA, BSc, B.Com, B.ED - 13 to 14 years) بیچلرز (بی اے، بی ایس سی، بی کام، بی ایڈ – تیرہ سے چودہ سال)<br>6 Master (MA, MSc, M.ED - 15 to 16 years) ماسٹرز (ایم اے، ایم ایس سی، ایم ایڈ – پندرہ سے سولہ سال)<br>7 MPhil, MS (17 to 18 years) ایم فل، ایم ایس (سترہ سے اٹھارہ سال)<br>8 Doctorate (> 18 years) ڈاکٹریٹ (اٹھارہ سے زیادہ سال))<br>99 Don't want to answer جواب نہیں دینا چاہتی/چاہتا
Q1.8 *(required)*	Q1.8 Marital status<br>ازدواجی حیثیت	1 Single غیر شادی شدہ<br>2 Married شادی شدہ<br>3 Divorced طلاق یافتہ<br>4 Separated علیحدگی<br>5 Widow بیوہ<br>96 Other: Please specify دیگر وضاحت کریں<br>99 Don't want to answer جواب نہیں دینا چاہتی / چاہتا
Q1.8a *(required)*	Q1.8a Other (Please specify)<br>دیگر وضاحت کریں<br>*Question relevant when: selected(${Q1.8} , '96')*	
Q1.9 *(required)*	Q1.9 Residence ownership<br>رہائش کی ملکیت	1 Rented کرائے پر<br>2 Owned اپنا گھر<br>96 Other: Please specify دیگر وضاحت کریں<br>99 Don't want to answer جواب نہیں دینا چاہتی/چاہتا
Q1.9a *(required)*	Q1.9a Other (Please specify)<br>دیگر وضاحت کریں<br>*Question relevant when: selected(${Q1.9} , '96')*	
Q1.10 *(required)*	Q1.10 How many members are in your household?<br>آپ کے گھر میں کتنے افراد ہیں؟	1 1-2<br>2 3-5<br>3 6-7<br>4 7+<br>99 Don't want to answer جواب نہیں دینا چاہتی/چاہتا
Q1.11 *(required)*	Q1.11 What kind of transport do you use?<br>آپ کس قسم کی سواری استعمال کرتے ہیں؟	1 Own car اپنی گاڑی<br>2 Own motorbike اپنا موٹرسائیکل<br>3 Public transport پبلک ٹرانسپورٹ<br>4 None of the above کوئی بھی نہیں<br>96 Other کوئی اور<br>99 Don't want to answer جواب نہیں دینا چاہتی / چاہتا
Q1.11a *(required)*	Q1.11a Other (Please specify)<br>دیگر وضاحت کریں<br>*Question relevant when: selected(${Q1.11} , '96')*	
Q1.12 *(required)*	Q1.12 Do you currently work?<br>کیا آپ کوئی کام کرتے ہیں؟	1 Self-employed اپنا کاروبار<br>2 Employed – private پرائیویٹ نوکری<br>3 Employed – government سرکاری ملازمت<br>4 Don't work بے روزگار<br>96 Other کوئی اور<br>99 Don't want to answer جواب نہیں دینا چاہتی/ چاہتا

Field	Question	Answer
Q1.12a *(required)*	Q1.12a Other (Please specify)<br>دیگر وضاحت کریں<br>*Question relevant when: selected(${Q1.12} , '96')*	
G1 > Risk Perceptions of COVID-19		
noteSec2	اب ہم آپ سے کرونا وائرس کے متعلق چند سوالات کریں گے	
Q2.1 *(required)*	Q2.1 Have you ever been infected with COVID-19?<br>کیا آپ کو کبھی کرونا وائرس ہوا ہے؟	1 Yes جی ہاں<br>2 No جی نہیں<br>98 Don't know پتہ نہیں<br>99 Don't want to answer جواب نہیں دینا چاہتی/چاہتا
Q2.2 *(required)*	Q2.2 How severe was the infection?<br>کتنا شدید ہوا تھا؟<br>*Question relevant when: selected(${Q2.1} , '1')*	1 No symptoms محسوس نہیں ہوا<br>2 Mild infection, managed at home معمولی سا محسوس ہوا، گھر پر علاج کیا<br>3 Serious illness, required hospitalization شدید متاثر ہوا، ہسپتال جانے کی ضرورت پڑی<br>4 Required ICU care کی ضرورت پڑی ICU<br>98 Not sure / Don't know معلوم نہیں<br>99 Don't want to answer جواب نہیں دینا چاہتی / چاہتا
Q2.3 *(required)*	Q2.3 Has someone in your household ever been infected with Covid-19?<br>کیا کبھی آپ کے گھر کے کسی فرد کو کرونا وائرس ہوا؟	1 Yes جی ہاں<br>2 No جی نہیں<br>98 Not sure/Don't know معلوم نہیں<br>99 Don't want to answer جواب نہیں دینا چاہتی/چاہتا
Q2.4 *(required)*	Q2.4 How do you think Coronavirus spreads?<br>آپ کے خیال میں کرونا وائرس کیسے پھیلتا ہے؟	1 It is not transmissible متعدی نہیں ہے<br>2 It is transmissible from person to person – physical contact متعدی ہے – ایک شخص سے دوسرے شخص کوچھونے سے لگ سکتا ہے<br>3 It is transmissible through sneezing/coughing (air borne) چھینکنے اور کھانسنے سے پھیلتا ہے<br>96 Other (Please specify) دیگر (براہ مہربانی وضاحت کریں))<br>98 Not sure/Don't know معلوم نہیں<br>99 Don't want to answer جواب نہیں دینا چاہتی/چاہتا
Q2.4a *(required)*	Q2.4a Other (Please specify)<br>دیگر وضاحت کریں<br>*Question relevant when: selected(${Q2.4} , '96')*	
Q2.5 *(required)*	Q2.5 How do you protect yourself from Coronavirus?<br>آپ کرونا وائرس سے اپنی حفاظت کیسے کرتے / کرتی ہیں؟	1 I Don't take any measures کوئی حفاظتی اقدامات نہیں لیتے<br>2 I keep distance from others دوسروں سے فاصلے رکھتے ہیں<br>3 I wear a mask when I go out باہر ماسک پہن کر جاتے/جاتی ہیں<br>4 I avoid large gatherings (indoor or outdoor) بڑے اجتماعات سے دور رہتے ہیں<br>5 Wash hands / use sanitizer regularly / باقاعدگی سے ہاتھ دھوتی ہوں یا سینیٹائزر کا استعمال کرتا / کرتی ہوں<br>96 Other (Please specify) دیگر (براہ مہربانی وضاحت کریں))<br>98 Not sure/Don't know معلوم نہیں<br>99 Don't want to answer جواب نہیں دینا چاہتی/چاہتا
Q2.5a *(required)*	Q2.5a Other (Please specify)<br>دیگر وضاحت کریں<br>*Question relevant when: selected(${Q2.5} , '96')*	

Field	Question	Answer
Q2.6 *(required)*	Q2.6 Are you worried that you will contract Coronavirus<br>کیا آپ کو اس بات کی فکر ہے کہ آپ کو کرونا وائرس ہو سکتا ہے؟	1 Strongly Agree مکمل طور پر متفق<br>2 Agree متفق<br>3 Neutral کوئی رائے نہیں ہے<br>4 Disagree غیر متفق<br>5 Strongly Disagree مکمل طور پر غیر متفق<br>98 Not sure/Don't know معلوم نہیں<br>99 Don't want to answer جواب نہیں دینا چاہتی/ چاہتا
Q2.7 *(required)*	Q2.7 Where do you fear is the most risk of getting COVID-19?<br>سب سے زیادہ آپ کو کہاں سے کرونا وائرس لگنے کا ڈر رہتا ہے؟	1 At home گھر سے<br>2 At work کام سے<br>3 During transport / to-fro ٹرانسپورٹ سے<br>4 Market places دکانوں سے<br>96 Other (Please Specify) دیگر وضاحت کریں<br>99 Don't want to answer جواب نہیں دینا چاہتی/چاہتا
Q2.7a *(required)*	Q2.7a Other (Please specify)<br>دیگر وضاحت کریں<br>*Question relevant when: selected(${Q2.7} , '96')*	
G1 > Vaccine Awareness and status		
noteSec3	اب ہم آپ سے کرونا کی ویکسین کے متعلق چند سوالات کریں گے	
Q3.1 *(required)*	Q3.1 In your opinion, has vaccination for COVID-19 begun in Pakistan?<br>کیا آپ کے خیال میں پاکستان میں کرونا وائرس کی ویکسینیشن شروع ہو چکی ہے؟	1 Yes جی ہاں<br>2 No جی نہیں<br>98 Not sure/don't know معلوم نہیں<br>99 Don't want to answer جواب نہیں دینا چاہتی/چاہتا
Q3.2 *(required)*	Q3.2 What are your sources of information for COVID-19 vaccination?<br>آپ کووڈ 19 ویکسینیشن کی معلومات کن زرائع سے حاصل کرتے ہیں؟	1 Television ٹیلی ویژن<br>2 Radio ریڈیو<br>3 Social Media Sites (including whatsapp) سوشل میڈیا (بشمول واٹس ایپ)<br>4 Government SMS / Calls حکومتی ایس ایم ایس اور کال<br>5 Newspapers اخبار<br>6 Family, friends and/or community members خاندان، دوست اور احباب<br>7 Doctors, medical experts or community health workers ڈاکٹر، میڈیکل اسپیشلسٹ، ہیلتھ ورکرز<br>8 Religious leaders مذہبی رہنما<br>9 Government officials سرکاری ملازم<br>96 Other (Specify) دیگر وضاحت کریں
Q3.2a *(required)*	Q3.2a Other (Please specify)<br>دیگر وضاحت کریں<br>*Question relevant when: selected(${Q3.2} , '96')*	
Q3.3 *(required)*	Q3.3 Do you know names of any specific vaccine?<br>کیا آپ کو کسی ویکسین کا نام معلوم ہے؟	1 Pfizer<br>2 Moderna<br>3 Sinopharm<br>4 CanSino<br>5 Sputnik<br>6 Sinovac<br>7 Oxford - AstraZeneca<br>8 Johnson & Johnson<br>9 All of the above سب کا پتہ ہے<br>10 Others (Specify) دیگر (وضاحت کریں)<br>98 Don't know the names نام نہیں معلوم<br>99 Don't want to answer جواب نہیں دینا چاہتی/ چاہتا
Q3.3a *(required)*	Q3.3a Other (Please specify)<br>دیگر وضاحت کریں<br>*Question relevant when: selected(${Q3.3} , '10')*	

Field	Question	Answer
Q3.4 *(required)*	Q3.4 Have you registered or received vaccination for Covid 19?<br>کیا آپ نے کرونا وائرس ویکسین کے لیے رجسٹر کیا ہے یا ویکسین لگوائی ہے؟	1 Yes, I have registered but have not been vaccinated ہاں، رجسٹریشن کروا لی ہے لیکن ویکسین نہیں لگی<br>2 No, I have not registered and not vaccinated نہیں، میں نے رجسٹریشن نہیں کروائی اور ویکسین بھی نہیں لگوائی<br>3 Yes, I have registered and received my first dose ہاں، میں نے رجسٹر کیا ہے اور ویکسین کا پہلا انجیکشن لگوا لیا ہے<br>4 Yes, I have registered and received my second dose ہاں، میں نے رجسٹر کیا ہے اور ویکسین کا دوسرا انجیکشن لگوا لیا ہے<br>98 Don't know/not sure معلوم نہیں<br>99 Don't want to answer جواب نہیں دینا چاہتی/چاہتا
Q3.5 *(required)*	Q3.5 Has anyone in your family registered for Covid-19 vaccination?<br>کیا آپ کے گھر کے کسی فرد نے کرونا کی ویکسین کے لیے رجسٹر کروایا ہے؟	1 Yes جی ہاں<br>2 No جی نہیں<br>3 Not applicable لاگو نہیں ہوتا<br>98 Don't know / not sure معلوم نہیں<br>99 Don't want to answer جواب نہیں دینا چاہتی / چاہتا
Q3.5.1 *(required)*	Q3.5.1 Has anyone in your family received atleast one dose of Covid-19 vaccine?<br>کیا آپ کے گھر کے کسی فرد نے کرونا کی ویکسین کا ایک بھی ٹیکہ لگوایا ہے؟	1 Yes جی ہاں<br>2 No جی نہیں<br>3 Not applicable لاگو نہیں ہوتا<br>98 Don't know / not sure معلوم نہیں<br>99 Don't want to answer جواب نہیں دینا چاہتی / چاہتا
Q3.6 *(required)*	Q3.6 How far is your nearest CVC (Vaccination Centre)?<br>آپ کے قریب ترین ویکسینیشن سنٹر آپ سے کتنے فاصلے پر ہیں؟	1 Less than one kilometre ایک کلومیٹر سے کم<br>2 1-2 kilometres ایک سے دو کلومیٹر<br>3 2+ kilometres دو کلومیٹر سے زیادہ<br>98 Don't know / Not sure معلوم نہیں<br>99 Don't want to answer جواب نہیں دینا چاہتی/چاہتا
Q3.7 *(required)*	Q3.7 Which country's vaccine do you think is safe and effective for use?<br>آپ کے خیال میں کونسے ملک کی ویکسین لگوانا محفوظ اور موثر ہے؟	1 China Manufactured چائنہ میں بنی ہوئی<br>2 Europe/US manufactured یورپ/امریکہ میں بنی ہوئی<br>3 UK manufactured برطانیہ میں بنی ہوئی<br>4 Russia-Manufactured روس میں بنی ہوئی<br>5 None of the above کوئی بھی نہیں<br>6 Don't know the countries of origin نہیں معلوم کہ کن ممالک میں ویکسین بن رہی ہے<br>7 Don't know which of these are safe and effective نہیں معلوم کہ ان میں سے کونسی ویکسین محفوظ اور موثر ہے<br>96 Other (Please Specify) دیگر وضاحت کریں<br>99 Don't want to answer جواب نہیں دینا چاہتی / چاہتا
Q3.7a *(required)*	Q3.7a Other (Please specify)<br>دیگر وضاحت کریں<br>*Question relevant when: selected(${Q3.7} , '96')*	
Q3.8 *(required)*	Q3.8 Will you consider getting vaccinated if a free of cost government administered vaccine is provided?<br>کیا آپ ویکسین لگوانا پسند کریں گے اگر حکومت آپ کو ویکسین مفت میں فراہم کرے؟	1 Strongly willing مکمل آمادہ<br>2 Willing کچھ حد تک آمادہ<br>3 Neutral کوئی رائے نہیں<br>4 Unwilling کچھ حد تک غیر آمادہ<br>5 Strongly unwilling بالکل غیر آمادہ<br>98 Don't know / not sure معلوم نہیں<br>99 Don't want to answer جواب نہیں دینا چاہتی / چاہتا

Field	Question	Answer
G1 > Health Care seeking behaviour		
noteSec4	اب ہم آپ سے آپکی صحت سے متعلق چند سوالات پوچھیں گے .	
Q4.1 *(required)*	Q4.1 How often do you visit a doctor/healthcare professional for general medical care?<br>آپ کتنی کثرت سے طبی معائنے یا علاج کے لیے ڈاکٹر کے پاس جاتے / جاتی ہیں؟	1 At least once a month (or more) کم سے کم مہینے میں ایک بار<br>2 Once a year (or less) سال میں ایک بار<br>3 In Emergency Situation صرف ایمرجنسی کی صورت حال میں<br>4 Never کبھی نہیں<br>99 Don't want to answer جواب نہیں دینا چاہتی / چاہتا
Q4.2 *(required)*	Q4.2 Did you seek treatment for your last illness?<br>کیا آپ نے اپنی آخری بیماری کے لیے علاج کروایا تھا؟	1 Yes جی ہاں<br>2 No جی نہیں<br>98 Don't know / Not sure معلوم نہیں<br>99 Don't want to answer جواب نہیں دینا چاہتی/چاہتا
Q4.3 *(required)*	Q4.3 Has any child in your household received routine immunization?<br>کیا آپ کے گھر کے کسی بچے کو حفاظتی ٹیکے لگے ہیں؟	1 Yes جی ہاں<br>2 No جی نہیں<br>3 Not applicable لاگو نہیں ہوتا<br>98 Don't know / not sure معلوم نہیں<br>99 Don't want to answer جواب نہیں دینا چاہتی / چاہتا
Q4.4 *(required)*	Q4.4 How do you consider the status of your health?<br>اپنی صحت کی صورت حال کے بارے میں آپکا کیا خیال ہے؟	1 I am healthier than most people I know میں اکثر لوگوں سے زیادہ صحت مند ہوں<br>2 I have average health میری صحت گزارے کے قابل ہے<br>3 I am occasionally unhealthy میں کبھی کبھی بیمار رہتا / رہتی ہوں<br>4 I have a chronic illness مجھے دائمی بیماری ہے<br>98 Don't know / not sure نہیں معلوم<br>99 Don't want to answer جواب نہیں دینا چاہتی/چاہتا
G1 > Social Services Awareness		
Q5.1 *(required)*	Q5.1 Which among these do you know about or are working in your area?<br>ان میں سے آپ کن کے بارے میں جانتے ہیں یا آپ کے علاقے میں کام کر رہی ہیں؟	1 NGOs این جی اوز<br>2 CBOs سی بی اوز<br>3 Private charitable Organizations نجی خیراتی ادارے<br>4 Government welfare institutes حکومتی فلاحی ادارے<br>5 None of the above کوئی نہیں<br>98 Don't know / not sure معلوم نہیں<br>99 Don't want to answer جواب نہیں دینا چاہتی/چاہتا
G1 > Campaign / Intervention Related Questions		
Q6.1 *(required)*	Q6.1 Have you received any information of COVID-19 vaccination in the last one month?<br>کیا آپ کو آخری ایک مہینے میں کووڈ 19 ویکسینیشن کے متعلق کوئی معلومات حاصل ہوئی ہیں؟	1 Yes جی ہاں<br>2 No جی نہیں<br>98 Don't know / Not sure معلوم نہیں<br>99 Don't want to answer جواب نہیں دینا چاہتی / چاہتا
Q6.2 *(required)*	Q6.2 From where did you get this information?<br>یہ معلومات آپ کو کہاں سے حاصل ہوئی ہیں؟<br>*Question relevant when: selected(${Q6.1} , '1')*	1 Television ٹیلی ویژن<br>2 Radio ریڈیو<br>3 Social media انٹرنیٹ<br>4 Government SMS / call حکومتی ایس ایم ایس / کال<br>5 Friends / family دوست اور رشتہ دار<br>6 Pamphlets / banners پمفلٹ اور بینر<br>7 Mosques مساجد<br>96 Other (Please Specify) دیگر وضاحت کریں<br>99 Don't want to answer جواب نہیں دینا چاہتی / چاہتا
Q6.2a *(required)*	Q6.2a Other (Please specify)<br>دیگر وضاحت کریں<br>*Question relevant when: selected(${Q6.2} , '96')*	

Field	Question	Answer
Q6.3 *(required)*	Q6.3 Did you see a mobile vaccination van in the past month?<br>کیا آپ کو پچھلے مہینے میں کوئی ویکسینیشن وین دکھائی دی ہے؟	1 Yes جی ہاں<br>2 No جی نہیں<br>98 Don't know / not sure معلوم نہیں<br>99 Don't want to answer جواب نہیں دینا چاہتی / چاہتا
Q6.4 *(required)*	Q6.4 Did you use that facility?<br>کیا آپ نے اس سہولت کا استعمال کیا؟<br>*Question relevant when: selected(${Q6.3} , '1')*	1 Yes جی ہاں<br>2 No جی نہیں<br>99 Don't want to answer جواب نہیں دینا چاہتی / چاہتا
contactno *(required)*	Respondent's contact number?<br>جواب دہندہ کا رابطہ نمبر ؟<br>*Type 9988 if Refused or NA اگر انکار کرے یا فون نہ ہو تو ۹۹۸۸ درج کریں*	
Q1.1_copy *(required)*	Q1.1 Name of the Interviewer<br>انٹرویو لینے والے کا نام<br>*Question relevant when: selected(${consent} , '2') or selected(${consent} , '3')*	
Q1.2.1_copy *(required)*	Q1.2.1 Location<br>*Question relevant when: selected(${consent} , '2') or selected(${consent} , '3')*	1 I-10<br>2 G-7 (66 Quarters)<br>3 F-7 (France Colony)<br>4 Bhara Kahu<br>5 Dhok Hassu
coord_copy *(required)*	Coordinates<br>*GPS coordinates can only be collected when outside.*<br>*Question relevant when: selected(${consent} , '2') or selected(${consent} , '3')*	
