# Supplementary material for: Community engagement to increase vaccine uptake: Quasi-experimental evidence from Islamabad and Rawalpindi, Pakistan
Source: PLoS One. 2022 Dec 1;17(12):e0274718. doi: 10.1371/journal.pone.0274718 (PMC9714835; doi:10.1371/journal.pone.0274718)
Supplement: Attachment — Submitted filename: Response to Reviewers.docx [file pone.0274718.s007.docx]

Dear Dr Harapan,

Thank you for the invitation to revise and resubmit the manuscript titled: ‘Community engagement to increase vaccine uptake: Quasi-experimental evidence from Islamabad and Rawalpindi, Pakistan’. We also thank the reviewers for their time and feedback on this manuscript. Their collective comments have prompted us to improve the manuscript substantially. Below we present a point-by-point response to the comments raised.

| **Comment** | **Response** |
| --- | --- |
| **Reviewer #1:** |  |
| This is an excellently written study, which takes advantage of a rigorously obtained sample and robust statistics. The message is important and timely. After reading the manuscript I have only a couple of minor points: | Thank you for taking out the time to review our manuscript. We present the response to your comments below referring to the Simple Markup Track Changes version of the manuscript. |
| line 58: I think "pregnant women" is more precise | We have replaced “women giving birth” with “pregnant women” (line 58). |
| line 60: I think "all the world's adult population" is more precise for the initial phase of the rollout | We agree and have corrected this now (line 60). |
| You mention a difference between acceptance of a vaccine and actual uptake, and explain it well through the TPB and difficulties in access. Could this also be due to some social desirability bias - of stating you were willing within a survey but then not really caring to get it? | Yes, this is possible as surveys were conducted through enumerators, and respondents might have shown more willingness than their actual intentions. We have addressed this in the Limitations section (lines 404-406). |
| **Reviewer #2:** |  |
| Thank you for the reviewing opportunity. Please find below my comments on the manuscript entitled “Community engagement to increase vaccine uptake: Quasi-experimental evidence from Islamabad and Rawalpindi, Pakistan”. The study is well-design but requires several clarifications. More importantly, this study has a significance reporting the influence of distance-to-vaccination site on vaccine uptake and personalized campaign (though authors still must clarify further what personalized campaign mean in their research). | Thank you for taking out the time to review our manuscript. We present the response to your comments below referring to the Simple Markup Track Changes version of the manuscript. |
| 1. Please pay attention to the grammar and punctuation. Many errors were found, I suggest author to improve the English of this manuscript. | We have improved the language of the manuscript. |
| 2. Line 47—48. As of?? Provide the date when the data were recorded. | We have included the date for cases and deaths (lines 48-49). |
| 3. After this, “…efficacy and safety of the vaccine. ” (Line 62), you may incorporate the following studies that reported on vaccine acceptance rate at different efficacy and safety levels:  i. Rosiello et al. Narra J 2021; 1(3): e55-doi: 10.52225/narra.v1i3.55 ii. Rayhan et al. Narra J 2022; 2(2): e85-doi: 10.52225/narra.v2i2.85 | Thank you for pointing out. We have referenced these articles as these seemed relevant (line 62). |
| 4. Line 73—77. How the data stated therein were collected? Like trust, how this was measured in the cited study? The government was shown to have negligent attitudes towards those who live in the slum area, how this was observed by the cited study? Authors have to make sure all statements are scientifically sound by checking the credibility and reliability of the data used in their statements. Please modify the paragraph. | We have modified the paragraph and cited relevant studies that discuss the attitude of governments towards informal urban settlements; the lack of trust derives from the governments’ perception of these communities as illegal (Lines 73-77). |
| 5. “..their low participation in the ongoing vaccination efforts.” Have the data been published? Please clarify. | The vaccination data was not disaggregated by localities but had city-level vaccination rates. Therefore, we could not use that. This argument was based on prior experience with other types of vaccinations (such as child immunization) in urban slums (lines 136-139). |
| 6. The method is already clear. But, some clarifications are needed: I. Can you please provide the translated version for Fig 1S? It’s a good information for those who want to replicate the same approach. II. Line 194 “…helped improve access of community…” Have been wondering what ‘access’ meant by authors. Could you please clarify? III. Control and treatment groups are from different localities? Please indicate so in Table 1. Also, what are the rationales? | I. Thank you for pointing this out. We have now included a translated version of the pamphlet in Supporting documents as well (S2 Fig) and mentioned this in line 184. II. We have removed this line as it was causing confusion with line 189-190. Improving access meant bringing COVID-19 Mobile Vaccination Camps (MVCs) to these underserved localities, while volunteers facilitated with the vaccination process on the days that MVCs were held. III. Our interventions were community-focused and not individual-focused, therefore to avoid contamination/spill-overs of intervention in the control group, we took different localities (explained in lines 143-146). |
| 7. Line 257. “Willingness to receive vaccines increased substantially..” Where can we see the data? Table 3? Please cite the table. | We have cited the table now (line 261). |
| 8. Line 309. “Living near a CVC was correlated with higher willingness..” Based on what parameter the correlation was drawn? | The reference category included people who did not know about the distance to the nearest CVC as they were residing very far to know the distance. “Living near a CVC was correlated with higher willingness” means that they were close enough to approximate the distances to the CVC. Hence, people living near a CVC include both less than 2 kms and 2+ kms. However, closer distances (less than 2 kms) were associated with higher uptake than somewhat further distances (2+ kms). We have clarified this in lines 312-315. |
| 9. Line 328, “engaging with community leaders, and building partnerships with local organizations” What results reflect this statement. I mean I hardly found the effect of the community leaders. And for the local organization, do you mean the NGO? Please use the same term for this issue to avoid confusion by readers. | These mean the results of our intervention (Table 4) and not the control variable that we have added in our models (Table 5). The control variable asked if any NGO/CBO was working in the area to access a respondents’ awareness level. However, here we talk about the specific NGOs/CBOs that we partnered with for our interventions. Similarly, the effects of community leaders come from the interventions (Table 4) and not the control variable of sources of information. We have clarified this by using the same terms in discussion (lines 331-333) as in our interventions section in the methodology (lines 182-183 and 191-192). |
| 10. Please clarify what personalized means is all about? Using local language? Or is there any other considerations for this. | Personalized means such interventions that are contextualized and includes raising awareness at community levels using printed material in local languages, engaging with community leaders, and building partnerships with local CBOs and NGOs, which we have explained in lines 332-334. This is in contrast to other means of awareness raising such as television or the internet which are non-personalized to communities (lines 336-337). |
| 11. Please comment on the context of culture, belief, and religion of the communities pertaining to the vaccine acceptance. Author may refer to this study: Hassan et al. Narra J. 2021; 1(3): e57—doi:10.52225/narra.v1i3.57 | We did not collect information on cultures and beliefs, however these can be assumed to be similar across our treatment and control groups, given the localities were similar – low-income and marginalized. We have included ethnicity in our models, with only Urdu speaking respondents, which were only a few, having significant change in vaccine uptake; all other ethnicities show similar willingness and uptake. |
